# A teenage patient with autosomal recessive polycystic kidney disease, a splenorenal shunt, and congenital hepatic fibrosis: a case report

**DOI:** 10.1590/2175-8239-JBN-2018-0081

**Published:** 2018-09-06

**Authors:** Vinicius Danieli Scarioti, Lucia Tabim de Oliveira, Anye Caroline Mattiello, Nayara dos Santos Gomes

**Affiliations:** 1 Hospital São José Departamento de Clínica Médica Jaraguá do SulSC Brasil Hospital São José, Departamento de Clínica Médica, Jaraguá do Sul, SC, Brasil.; 2 Hospital São José Departamento de Nefrologia Jaraguá do SulSC Brasil Hospital São José, Departamento de Nefrologia, Jaraguá do Sul, SC, Brasil.

**Keywords:** Polycystic Kidney, Autosomal Recessive, Liver Cirrhosis, Adolescent

## Abstract

A 16-year-old female patient previously diagnosed with autosomal recessive polycystic kidney disease (ARPKD) presented with acute bilateral pneumonia, upper gastrointestinal bleeding caused by ruptured esophageal varices, ascites, and lower limb edema. She required intensive care and an endoscopic procedure to treat the gastrointestinal bleeding. The analysis of the differential diagnosis for chronic liver disease indicated she had a spontaneous splenorenal shunt. Ultrasound-guided biopsy revealed the patient had cirrhosis, as characteristically seen in individuals with ARPKD. She had no symptoms at discharge and was referred for review for a combined transplant.

## INTRODUCTION

ARPKD occurs in approximately 1:20,000 live births.^1^ Although less frequent than the dominant form of the disease, it is a common inherited ciliopathy caused by mutations on gene PKD1.^2^ It may involve a number of systems and requires multidisciplinary care. In addition to polycystic kidney disease, liver fibrosis is a nearly universal finding at birth, since one of the proteins expressed by PKD1 is fibrocystin/polyductin, present in the renal tubules (particularly in the collecting duct and thick ascending limb of Henle's loop), bile ducts in the liver, and pancreatic ducts.^3^ These biliary alterations may also induce the dilatation of the biliary tree and the onset of Caroli syndrome, a condition frequently observed in patients with liver fibrosis that predisposes them to having repetition cholangitis by biliary stasis.^4^ Along with ruptured esophageal varices, these conditions comprise the main potentially fatal complications in childhood and adult age.^2^

## CASE REPORT

A.P.A., a 16-year-old female student born and residing in Jaraguá do Sul, SC, Brazil, arrived at the emergency unit after suffering from dry cough and dyspnea for four days, along with hematemesis and bloody diarrhea. She said she had been diagnosed with polycystic kidney disease as a child and that a pediatric nephrologist was treating her. The patient also mentioned that she had been referred for a kidney transplant on account of chronic kidney disease and that she was being treated for systemic hypertension and anemia secondary to renal impairment. Physical examination revealed she was pale, rational, attentive, responsive, and oriented. Her blood pressure (BP) was 140x70 mmHg. Lung auscultation showed she had vesicular breath sounds and basilar crackles on her right lung. Heart auscultation showed a regular rhythm with two sounds and no murmur. She had tachycardia (140 beats per minute), tachypnea (respiratory rate [RR] 20 breaths per minute), a painless abdomen with a palpable spleen, and no edema on her legs. Tests performed three months prior to admission showed her hemoglobin and creatinine levels had been at 8.0 g/dL and 2.5 mg/dL, respectively (creatinine clearance 32.21 mL/min estimated by the Cockroft-Gault formula). Tests performed on admission read as follows: hemoglobin 5.8 g/dL; hematocrit 15.9%; leukocytes 22,500 without a left shift; platelets 122,000; creatinine 5.39 mg/dL (creatinine clearance 14.94 mL/min); urea 158 mg/dL; C-reactive protein 81.8 U/L; serum sodium 141 mEq/L; and serum potassium 3.9 mEq/L. Chest X-ray findings were consistent with consolidation of the right upper lobe and lung infiltration on the left middle and lower zones. She was started on ceftriaxone, hydration, and received two bags of packed red blood cells.

Her respiratory pattern deteriorated on the first day of hospitalization. She became more tachypneic (RR: 36 breaths per minute) and presented stertor bilaterally down to the middle zone of her lungs, coffee ground vomitus, and early-stage edema in her lower limbs (2+/4+). She was referred to the ICU and was prescribed clarithromycin, oseltamivir, pantoprazole, and was placed on noninvasive ventilation. Point-of-care ultrasound examination showed B-lines in all lung areas bilaterally, no pleural effusion, and a fixed dilated inferior vena cava with no variation on ventilation. Her echocardiogram showed a left ventricle on the parasternal short axis with good contractility and no significant pericardial effusion on subjective evaluation.

**Figure 1:**
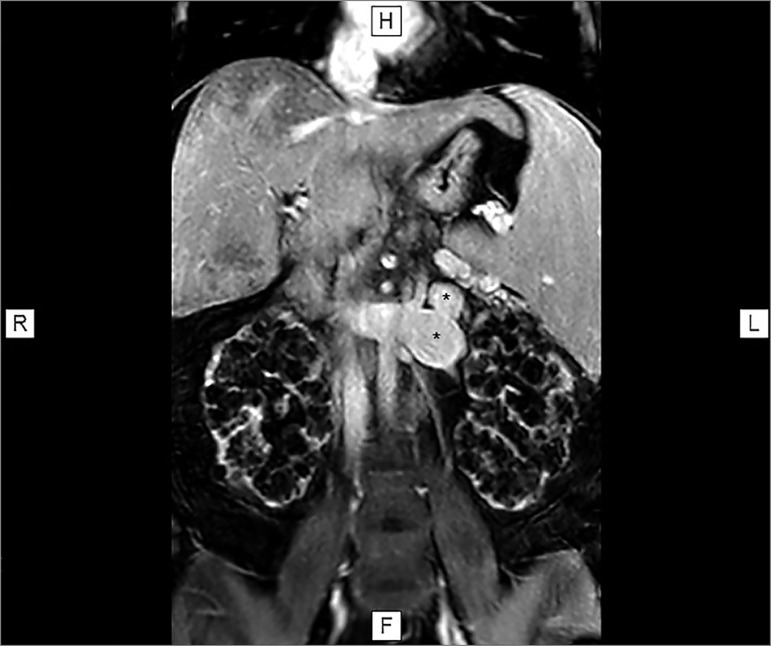
Upper abdomen MRA showing signs of chronic liver disease, polycystic kidneys, and a shunt between the left renal vein and a collateral vessel originating in the splenic vein (*).

She stayed in the ICU for eight days with episodes of melena and hematemesis. Endoscopic rubber band ligation was performed on three esophageal varices, and later another three medium-caliber varices were treated with sclerotherapy. Total abdomen ultrasound examination and other imaging tests revealed signs of chronic liver disease, major homogeneous splenomegaly, and a portal system without signs of thrombosis and minor flow velocity alterations. Magnetic resonance angiography (MRA) images of the upper abdomen showed a left splenorenal venous shunt and a significantly distended left renal vein draining into the inferior vena cava, which was also distended above the junction with the left renal vein. The patient had moderate ascites around the liver and spleen, in the paracolic gutters, and between intestinal loops (Figure 1). With the exception of the first days on the ICU, when she had hypoalbuminemia and thrombocytopenia and needed platelet replacement, her lab tests did not show significant liver dysfunction. All other liver function tests were normal.

**Figure 2:**
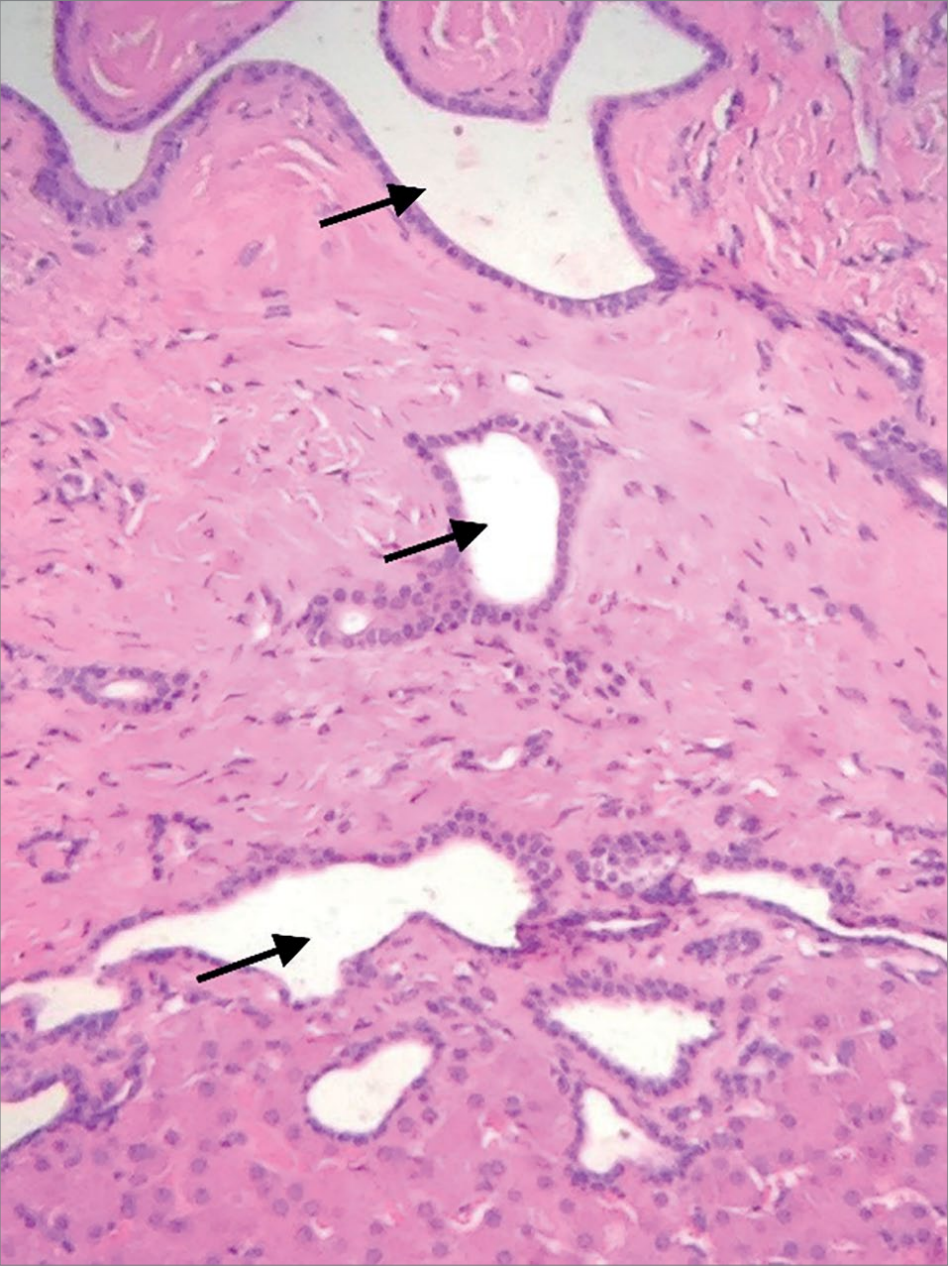
Hematoxylin and eosin (H&E) staining - 100x - Ducts with cysts (arrows) covered by cuboidal epithelial cells.

**Figure 3:**
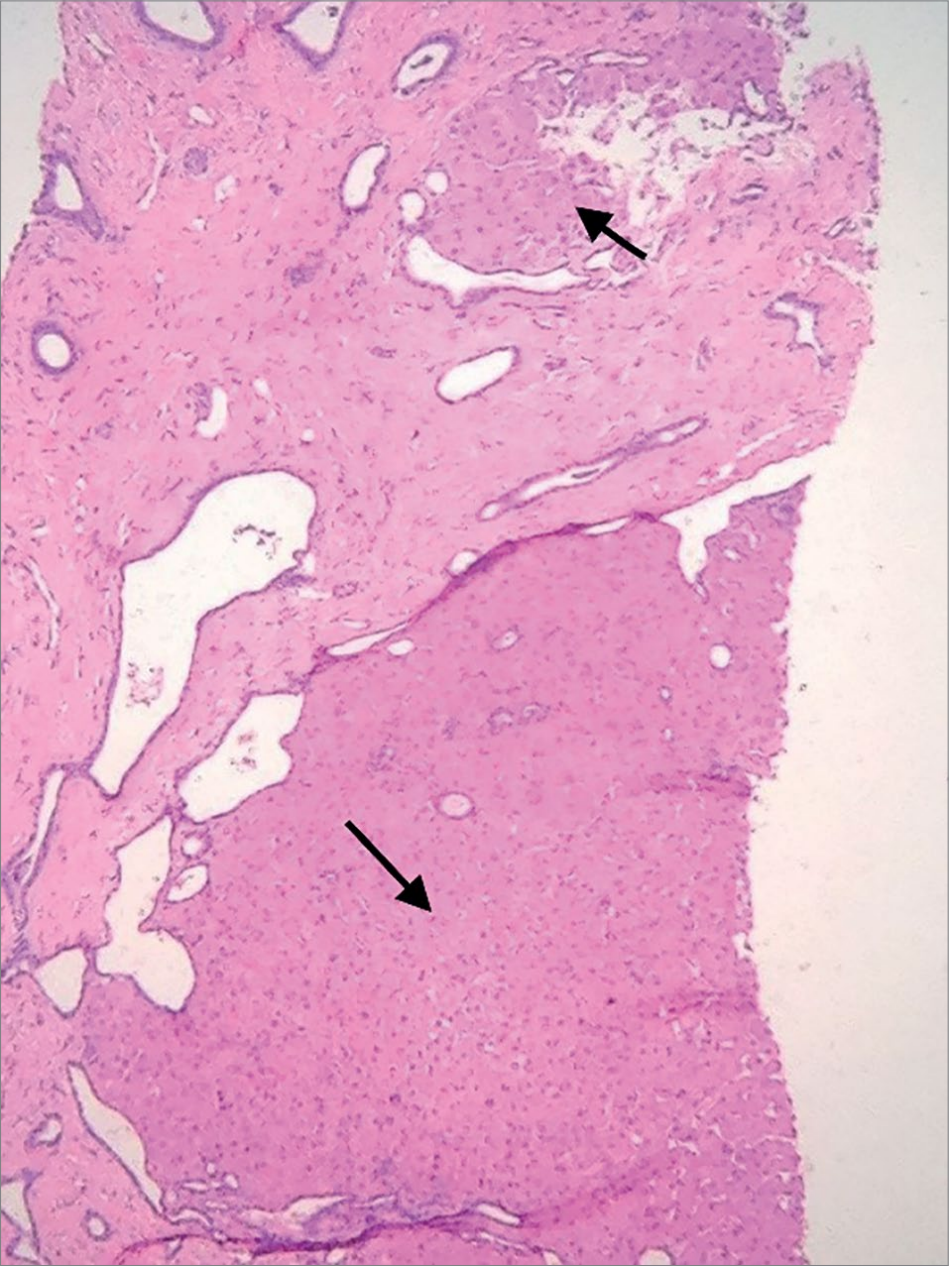
H&E - 40x - Liver cirrhosis defined by large fibrous septa delimiting residual parenchymal nodules (arrows).

The patient underwent further examination for chronic liver disease after she left the ICU, and her test results came back normal or negative. Her renal function kept on being monitored and her serum creatinine level stabilized around 2.5 mg/dL. Her 24-hour urinary protein was 130 mg and urine output was normal. She did not require renal replacement therapy. The patient underwent an ultrasound-guided biopsy of the liver and was diagnosed with diffuse liver fibrosis and cystic dilatation of the bile ducts (Figure 2), signs characteristically seen in liver cirrhosis (Figure 3). She was asymptomatic at discharge, without new episodes of bleeding, ascites or lower limb edema. Her BP was under control and she was prescribed an angiotensin-converting-enzyme inhibitor, a potassium-sparing diuretic, and a prophylactic nonselective beta-blocker for her esophageal varices. She was referred to the nephrology and transplant departments for additional evaluation.

## DISCUSSION

ARPKD is a severe form of polycystic kidney disease and a significant cause of morbidity and mortality in children.^5^ More than 300 mutations of gene PKD1 have been mapped, and no correlation has been established between genotypes and phenotypes;^6^ therefore, the genetic test used to confirm the presence of the disease - expensive and scarcely available - is not required in typical cases.^1^

Prenatal ultrasound alterations seen in the second trimester may suggest renal involvement when oligohydramnios, increased echogenicity, enlarged kidneys, and large cysts (> 10 mm) are found. Although they are not specific diagnostic findings, patients with these signs are required to undergo ultrasound examination every two to three weeks until the baby is born. Pulmonary hypoplasia caused by thoracic compression and oligohydramnios secondary to nephromegaly has been described as the main cause of death at birth. Other complications include respiratory distress and pneumothorax.^1^^,^^2^^,^^4^^,^^6^ However, there is no description in the literature of these patients being more prone to lung infections in childhood or adult age.

Nearly all patients with renal involvement at birth or during childhood have significant systemic hypertension. They require adequate management of their BP with medication and diet, and tend to improve with age.^1^^,^^6^

Liver alterations and some degree of fibrosis are present at birth in nearly all cases, leading to progressive increases in portal pressure and the onset of related clinical complications.^3^ Prophylactic use of nonselective beta-blockers such as propranolol to treat bleeding esophageal varices in children is not supported by the literature, and should generally not be indicated to such end.^2^ Some authors have suggested that individuals with ARPKD are at higher risk for liver cancer,^7^ but most agree that screening should be reserved for individuals aged 40+ years and that a definitive correlation cannot be established with the data available today.^1^^-^4

There is no other description in the literature of a case of spontaneous splenorenal shunt in an individual with ARPKD. The shunt stems from increased pressure in the portal vein, which ends up diverting blood flow to less resistant collateral vessels, a finding suggestive of worse liver function without impact on mortality.^8^ Several authors recommend splenorenal shunt surgery to alleviate pressure in the portal vein.^9^^,^^10^

There is no clinical cure for ARPKD other than a combined liver-kidney transplant (CLKT).^1^ There is doubt as to when and how to intervene on patients progressing to end-stage renal disease and overt liver cirrhosis,^11^ since little or no change is found in liver lab tests.^2^ CLKT is seldom performed in children - ten to thirty procedures are carried out every year in the world.^11^ The main indications are primary hyperoxaluria and ARPKD.^12^ Kidney transplant tends to be the preferred option, since mortality is greater among patients offered CLKT. Patients with refractory complications secondary to portal hypertension and/or recurrent cholangitis are preferentially offered CLKT.^11^

The clinical manifestations tied to ARPKD vary significantly. Extrarenal events are quite common in patients surviving neonatal life. Enhanced understanding of the formation of cystic structures is required to further the development of therapies designed to contain the progression of these anomalies. Finally, the significant levels of morbidity and mortality introduced by this condition call for more studies on the management and follow-up of a group of patients in need of multidisciplinary care.
